# Association between serum uric acid-to-high-density lipoprotein cholesterol ratio and metabolic dysfunction-associated steatotic liver disease among Chinese children with obesity

**DOI:** 10.3389/fendo.2024.1474384

**Published:** 2025-01-08

**Authors:** Meijuan Liu, Bingyan Cao, Qipeng Luo, Yanning Song, Kai Liu, Di Wu

**Affiliations:** ^1^ Department of Endocrinology, Genetics and Metabolism, Beijing Children’s Hospital, Capital Medical University, National Center for Children’s Health, Beijing, China; ^2^ Department of Pain Medicine, Peking University Third Hospital, Beijing, China

**Keywords:** children, uric acid-to-high-density lipoprotein cholesterol ratio, metabolic dysfunction-associated steatotic liver disease, obesity, risk

## Abstract

**Background:**

Metabolic dysfunction-associated steatotic liver disease (MASLD) has become one of the most prevalent chronic liver diseases worldwide. The serum uric acid-to-high-density lipoprotein cholesterol ratio (UHR) has been recognized as a novel marker for metabolic diseases, including MASLD. However, all previous studies were performed in adults.

**Objectives:**

To explore the relationship between the UHR and MASLD in Chinese children with obesity.

**Methods:**

A retrospective study was conducted including 1284 obese children hospitalized at Beijing Children’s Hospital between January 2016 and December 2022. Logistic regression analysis and restricted cubic splines were performed to assess the association between the UHR and the odds of MASLD. The receiver operator characteristic (ROC) curve analysis was used to estimate the diagnostic value of UHR for MASLD in children with obesity.

**Results:**

The prevalence of MASLD was high, which reached 61.76% in children with obesity. UHR levels were higher in obese children with MASLD than those with non-MASLD for both genders. After dividing all individuals into three groups according to the tertiles of UHR, the prevalence rate of MASLD increased progressively from the tertile 1 to tertile 3 of UHR (34.11% *vs*. 70.56% *vs*. 80.61%). Logistic regression analysis showed that obese children with higher UHR levels were significantly associated with MASLD risk, independent of confounding factors such as age, gender, body mass index (BMI), fasting blood glucose (FBG), alanine aminotransferase (ALT), aspartate aminotransferase (AST), gamma-glutamyl transferase (GGT), low-density lipoprotein cholesterol (LDL-C), triglycerides (TG), and creatinine (Cr). The non-linear relationship analysis demonstrated that a UHR between approximately 300 and 900 suggested a saturation effect of MASLD risk. ROC analysis indicated that UHR might serve as a predictive marker for diagnosing MASLD in obese children.

**Conclusions:**

In children with obesity, UHR is significantly associated with MASLD and might serve as a novel and useful predictor for MASLD onset.

## Introduction

Metabolic dysfunction-associated steatotic liver disease (MASLD), previously termed non-alcoholic fatty liver disease (NAFLD), is defined as steatotic liver disease that occurs in the context of metabolic dysfunction and the absence of harmful alcohol intake. This condition has gained increasing attention due to its rising prevalence, particularly in conjunction with the global obesity epidemic (1). The incidence of MASLD among children and adolescents is also rising, with prevalence estimates ranging from 3% to 11% in the USA (2), and approximately 7.01% in Asia (3). The proportion of children with obesity is even higher, approximately 52.49% of obese children suffer from MASLD (3). MASLD not only causes a wide spectrum of liver damage, ranging from simple steatosis to cirrhosis (4), but also contributes to many chronic diseases such as cardiovascular disease (5), chronic kidney disease (6), and cancers (7). Currently, liver biopsy is the gold standard for diagnosis of MASLD, but its clinical application is limited due to its invasiveness and high cost. Ultrasonography and computed tomography (CT) are frequently used to diagnose MASLD in clinical practice, however, the sensitivity and accuracy of ultrasonography are relatively low, and CT has a risk of radiation exposure (8). Therefore, seeking for noninvasive and convenient serum biomarker for early diagnosis of MASLD has gradually become a research direction.

Uric acid (UA), the main product of purine metabolism, was found to be closely associated with obesity and its related metabolic disorders (9–11). High-density lipoprotein cholesterol (HDL-C) is a plasma lipoprotein with excellent anti-inflammatory and antioxidant roles, which performs important functions in metabolic disorders (12). Emerging evidence demonstrates that both elevated UA and reduced HDL-C levels are linked to a higher risk of developing MASLD (11, 13). Previous studies by Zhou et al. found that hyperuricemia ranked first in pediatric MASLD comorbidities, followed by dyslipidemia (14). Recently, UA to HDL-C ratio (UHR) has attracted increasing attention as a valuable biomarker for cardiovascular disease (15, 16), insulin resistance (17, 18), and metabolic syndrome (19). Furthermore, some studies have found a strong relationship between UHR and MASLD (17, 20–26). In a cross-sectional study including 3766 American individuals, UHR was found to be independently related to an increased MASLD risk and the severity of liver steatosis (23). However, all previous studies were performed in adults, and no data was available for the association between UHR and MASLD in children. Therefore, this study aimed to investigate the relationship between UHR and MASLD risk in children with obesity.

## Methods

### Study population

A retrospective study was conducted among 1284 children with obesity who were hospitalized in the Department of Endocrinology, Genetics and Metabolism, Beijing Children’s Hospital (Beijing, China) from January 2016 to December 2022. Obesity was defined as body mass index (BMI) ≥95th percentile of children of the same age and gender according to the Centers for Disease Control and Prevention (CDC) standards] (27). Exclusion criteria were as follows: (1) children with systemic or organic diseases; (2) children taking UA-lowering drugs or lipid-lowering drugs; (3) children missing anthropometric or laboratory data. Ultimately, 1284 obese children (848 boys and 436 girls) were enrolled in this study. This study was approved by the ethics committee of Beijing Children’s Hospital, Capital Medical University. Written and informed consent was gained from all children and their families.

### Anthropometric measurements and laboratory examinations

The anthropometric measurements such as body weight, height, waist circumstance (WC), and hip circumference (HC) were measured following the standardized approaches. Body weight and height were measured with children dressed indoor clothing and without shoes. WC was measured by a non-extensible measuring tape placed between the top of the iliac crest and the lowest costal margin. HC was measured at the horizontal level of the widest portion of the buttocks using the same tape. BMI was calculated as the weight (in kg) divided by height (in m^2^).

Overnight fasting blood samples were collected for laboratory examinations, including fasting blood glucose (FBG), alanine aminotransferase (ALT), aspartate aminotransferase (AST), gamma-glutamyl transferase (GGT), creatinine (Cr), UA, total cholesterol (TC), triglycerides (TG), HDL-C, and low-density lipoprotein cholesterol (LDL-C). UHR was defined as UA (umol/L)/HDL-C (mmol/L). Liver B-ultrasound was performed for each participant by trained sonographers. The diagnosis of MASLD was based on the consensus of the Chinese Society of Pediatric Endocrinology and Metabolism (28).

### Statistical analyses

Data analysis was conducted using R software version 4.4.0 for Windows (R Foundation for Statistical Computing, Vienna, Austria). Quantitative variables were shown as means ± standard deviations (SD) and analyzed by the independent t-test. Logistic regression analysis was performed to investigate the association of UHR tertiles with the risk of MASLD, and the unadjusted and adjusted odds ratios (ORs) as well as 95% confidence intervals (CIs) were calculated. Adjustments were made for various confounding factors, including age, gender, BMI, FBG, ALT, AST, GGT, LDL-C, TG, and Cr. The receiver operator characteristic (ROC) curve analysis was applied to evaluate the diagnostic effectiveness of UHR for MASLD in children with obesity. Restricted cubic splines with 5 knots were applied to delineate the curve of associations between baseline UHR levels and the risk of MASLD, blue shading indicates the 95% confidence intervals around the estimates, and two cutoffs from restricted cubic spline curve were made concerning the risk of MASLD, because their ORs of the corresponding lower limit of 95% CI were calculated as 1.0. *P*<0.05 was considered to indicate statistical significance.

## Results

### Characteristics of the study participants

**Figure 1:**
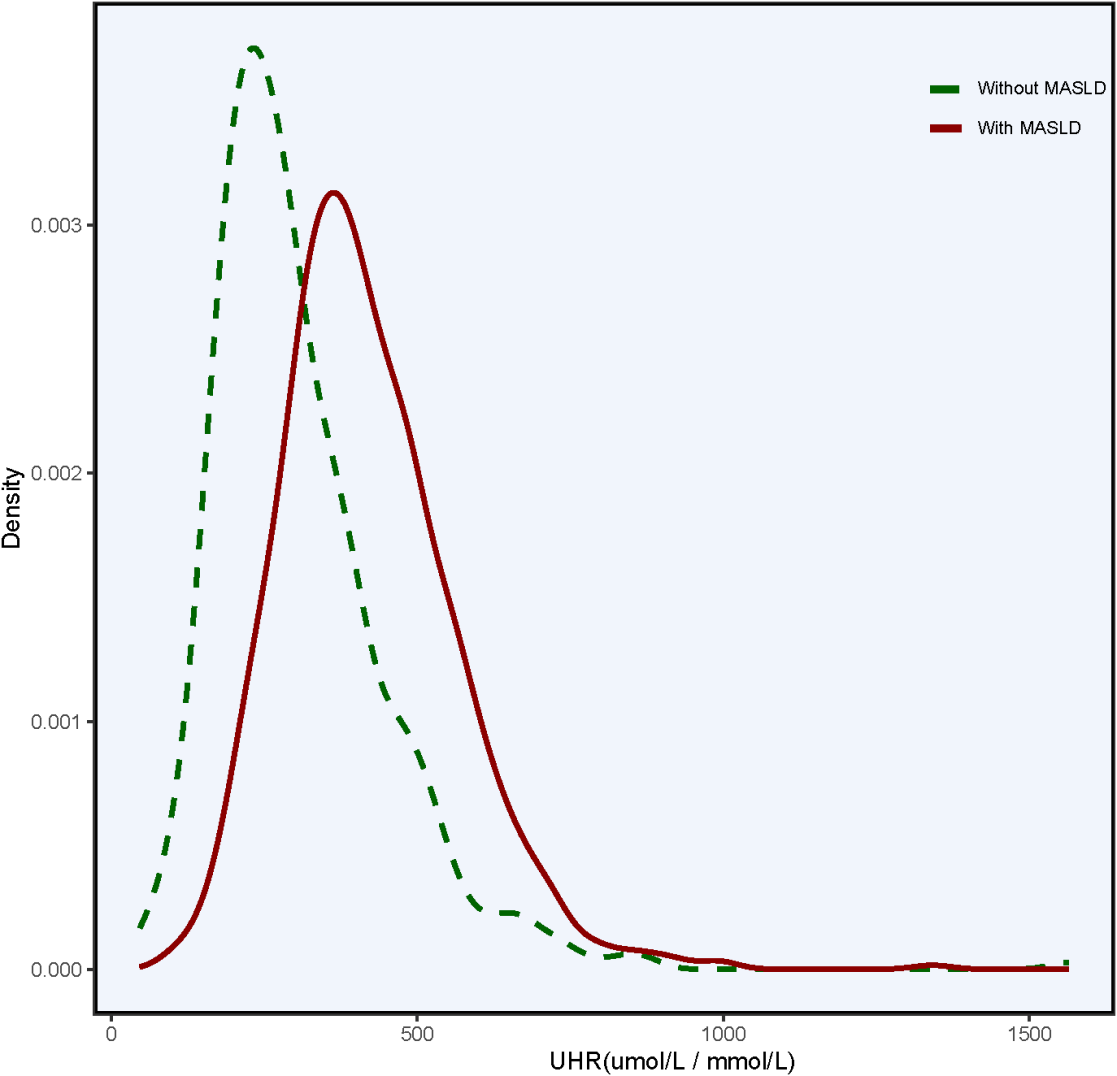
Kernel density plots showing the distribution of UHR. UHR, uric acid-to-high-density lipoprotein cholesterol ratio.

**Table 1** describes the baseline characteristics of the 1284 children with obesity depending on the status of MASLD. In both genders, obese children in the MASLD group were older than in the non-MASLD group. Meanwhile, they had higher BMI, BMI-Zscore, WC, HC, FBG, ALT, AST, GGT, TC, TG, LDL-C, Cr, and UA, but lower HDL-C values. Interestingly, UHR levels were higher in obese children with MASLD than those with non-MASLD for both genders (*P* all <0.05). As illustrated in **Figure 1**, a significant “rightward shift” of UHR was observed in the MASLD group compared with that in the non-MASLD group.

**Table 1:** Baseline characteristics of study subjects and the differences of factors between obese children with non-MASLD and MASLD.

Characteristics	Total (n=1284)		Boys (n=848)		Girls (n=436)	
	Non-MASLD (n=491)	MASLD (n=793)	Non-MASLD (n=270)	MASLD (n=578)	Non-MASLD (n=221)	MASLD (n=215)
Age (years)	9.61 ± 3.23	12.3 ± 2.42^a^	10.6 ± 3.19	12.4 ± 2.38^a^	8.42 ± 2.87	12.1 ± 2.51^a^
BMI (kg/m^2^)	25.9 ± 5.66	30.7 ± 4.79^a^	26.8 ± 5.01	30.7 ± 4.74^a^	24.7 ± 6.19	30.8 ± 4.92^a^
BMI-Zscore	2.10 ± 0.39	2.22 ± 0.37^a^	2.13 ± 0.43	2.24 ± 0.40^a^	2.07 ± 0.32	2.17 ± 0.29^a^
WC (cm)	88.1 ± 14.1	98.5 ± 11.0^a^	89.5 ± 13.6	99.5 ± 11.1^a^	84.9 ± 14.8	96.1 ± 10.6^a^
HC (cm)	94.6 ± 14.8	104.0 ± 11.4^a^	95.5 ± 14.5	104 ± 10.5^a^	92.7 ± 15.3	104 ± 13.6^a^
FBG (mmol/L)	6.01 ± 3.29	6.87 ± 3.62^a^	6.34 ± 3.91	6.84 ± 3.73^a^	5.61 ± 2.28	6.96 ± 3.31^a^
ALT (U/L)	25.0 ± 26.0	63.2 ± 68.9^a^	29.0 ± 30.4	64.9 ± 71.6^a^	20.2 ± 18.3	58.5 ± 60.9^a^
AST (U/L)	27.3 ± 19.4	43.4 ± 45.7^a^	28.1 ± 18.4	43.9 ± 48.5^a^	26.3 ± 20.4	42.0 ± 37.2^a^
GGT (U/L)	20.9 ± 13.9	38.6 ± 30.3^a^	23.8 ± 16.3	39.5 ± 30.8^a^	17.4 ± 9.04	36.3 ± 28.7^a^
TC (mmol/L)	4.28 ± 1.06	4.44 ± 1.02^a^	4.30 ± 1.16	4.41 ± 1.01^a^	4.25 ± 0.94	4.53 ± 1.03^a^
TG (mmol/L)	1.20 ± 1.15	1.59 ± 1.16^a^	1.24 ± 0.75	1.59 ± 1.19^a^	1.14 ± 1.51	1.61 ± 1.09^a^
HDL-C (mmol/L)	1.29 ± 0.31	1.12 ± 0.25^a^	1.28 ± 0.32	1.13 ± 0.26^a^	1.32 ± 0.30	1.11 ± 0.21^a^
LDL-C (mmol/L)	2.42 ± 0.70	2.76 ± 0.82^a^	2.44 ± 0.67	2.72 ± 0.81^a^	2.400.74	2.86 ± 0.86^a^
Cr (umol/L)	42.0 ± 12.2	47.1 ± 23.3^a^	44.5 ± 13.3	48.3 ± 26.6^a^	39.0 ± 9.95	44.0 ± 9.56^a^
UA (umol/L)	367 ± 112	447 ± 105^a^	391 ± 118	458 ± 110 ^a^	339 ± 96.1	418 ± 86.7^a^
UHR(umol/L/mmol/L)	307 ± 145	419 ± 142^a^	331 ± 146	430 ± 151 ^a^	227 ± 139	390 ± 111^a^

MASLD, metabolic dysfunction-associated steatotic liver disease; BMI, body mass index; WC, waist circumference; HC, hip circumference; FBG, fasting blood glucose; ALT, alanine aminotransferase; AST, aspartate transferase; GGT, glutamyl transpeptidase; TC, total cholesterol; TG, triglycerides; HDL-C, high-density lipoprotein- cholesterol; LDL-C, low-density lipoprotein- cholesterol; Cr, creatinine; UA, uric acid; UHR, uric acid to high-density lipoprotein-cholesterol ratio. ^a^ *P*< 0.05 referred to the comparison between non-MASLD and NAFLD.

### Prevalence of MASLD according to the UHR tertiles

**Figure 2:**
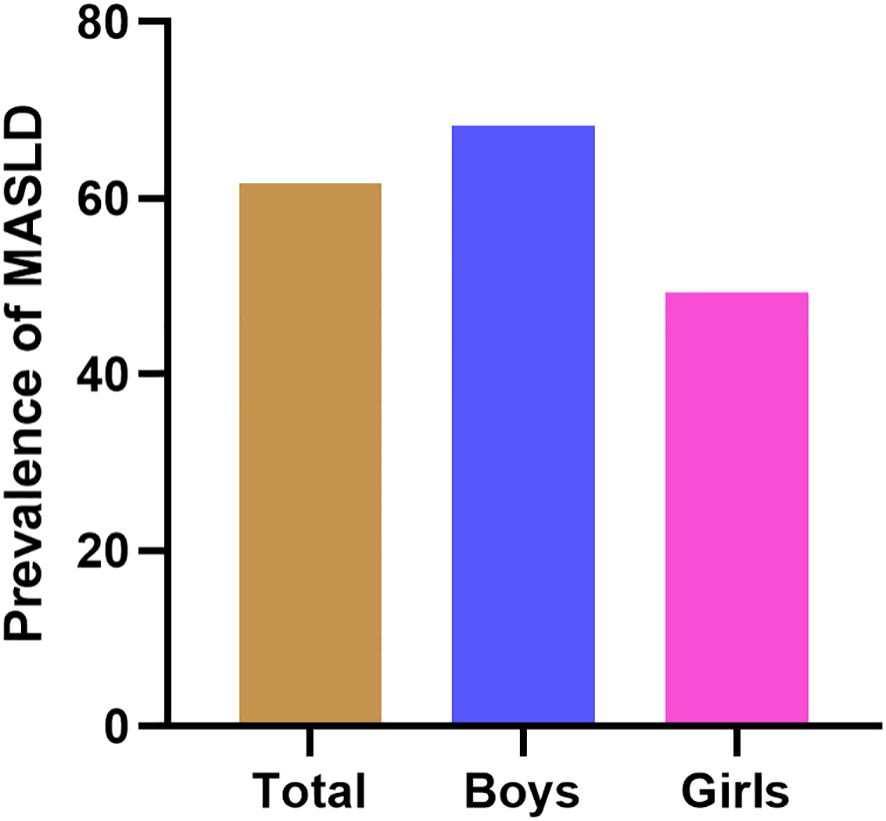
The prevalence of MASLD in children with obesity of different genders. MASLD, metabolic dysfunction-associated steatotic liver disease.

**Figure 3:**
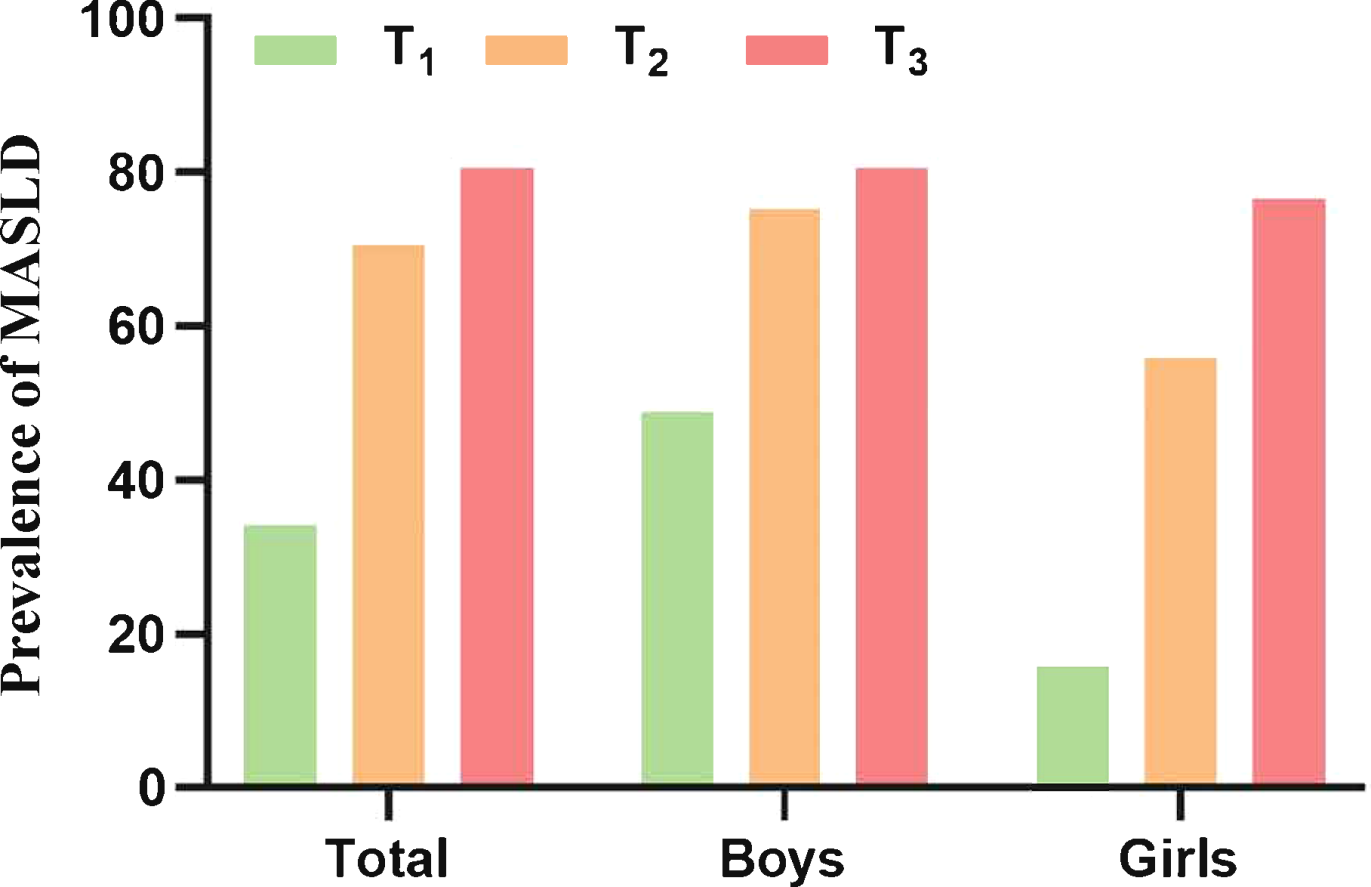
The prevalence of MASLD in children with obesity according to the UHR tertiles. UHR, uric acid-to-high-density lipoprotein cholesterol ratio; MASLD, metabolic dysfunction-associated steatotic liver disease.

As presented in **Figure 2**, the total prevalence of MASLD reached 61.76% in children with obesity, and the prevalence in boys was higher than that in girls (68.16% *vs*. 49.31%). In addition, after dividing all individuals into three groups according to the tertiles of UHR, the prevalence of MASLD was positively correlated with the UHR levels (**Figure 2**). The prevalence of MASLD was higher in UHR tertile 2 (70.56%), and even higher in UHR tertile 3 (80.61%) when compared with UHR tertile 1 (34.11%). This phenomenon was found among both genders and was more pronounced in girls. The prevalence of MASLD in UHR tertile 2 and tertile 3 was nearly 2.5 times and 3.9 times higher than in tertile 1, respectively (**Figure 3**).

### Correlation between UHR and the MASLD risk

To further investigate the correlation between UHR and the MASLD risk, univariate and multivariate logistic regression analyses were conducted. As shown in **Table 2**, UHR was positively associated with the MASLD risks in all three models: crude model (OR = 2.65, 95% CI: 2.26, 3.10), model 1 (OR = 1.82, 95% CI: 1.54, 2.15), and model 2 (OR = 1.52, 95% CI: 1.27, 1.81) (*P* all <0.05). Additionally, after dividing all children into three groups according to the tertiles of UHR, the unadjusted ORs for MASLD in the tertile 2 group and tertile 3 group were 4.66 (95% CI: 3.49, 6.22) and 8.08 (95% CI: 5.92, 11.05) compared with tertile 1. After adjusting for age and gender (Model 1), the ORs for MASLD in the tertile 2 and tertile 3 groups were 3.08 (95% CI: 2.26, 4.20) and 3.99 (95% CI: 2.83, 5.63). Further after adjusting for age, gender, BMI, FBG, ALT, AST, GGT, LDL-C, TG, and Cr (Model 2), the ORs of MASLD remained significantly increased for tertile 2 (OR = 2.51, 95% CI: 1.78, 3.54) and tertile 3 (OR = 2.79, 95% CI: 1.89, 4.12). In the analyses stratified by gender, the significantly higher ORs of MASLD in the tertile 2 and tertile 3 groups were observed in both genders, not only in the crude model but also in the adjusted models.

**Table 2:** Association of the MASLD with UHR levels.

	Crude model		Model 1		Model 2	
	OR (95% CI)	*P*	OR (95% CI)	*P*	OR (95% CI)	*P*
Total						
UHR (per SD increase)	2.65 (2.26, 3.10)	<0.01	1.82 (1.54, 2.15)	<0.01	1.52 (1.27, 1.81)	<0.01
UHR (tertile)						
Q1	Reference		Reference		Reference	
Q2	4.66 (3.49, 6.22)	<0.01	3.08 (2.26, 4.20)	<0.01	2.51 (1.78, 3.54)	<0.01
Q3	8.08 (5.92, 11.05)	<0.01	3.99 (2.83, 5.63)	<0.01	2.79 (1.89, 4.12)	<0.01
Boys						
UHR (per SD increase)	2.14 (1.79,2.57)	<0.01	1.76 (1.45,2.13)	<0.01	1.44 (1.16,1.78)	<0.01
UHR (tertile)						
Q1	Reference		Reference		Reference	
Q2	3.42 (2.37,4.95)	<0.01	2.58 (1.75,3.79)	<0.01	1.96 (1.28,3)	<0.01
Q3	5.81 (3.98,8.49)	<0.01	3.86 (2.56,5.82)	<0.01	2.52 (1.58,4.02)	<0.01
Girls						
UHR (per SD increase)	3.85 (2.79,5.33)	<0.01	2.22 (1.56,3.15)	<0.01	1.87 (1.29,2.71)	<0.01
UHR (tertile)						
Q1	Reference		Reference		Reference	
Q2	7.01 (4.25,11.55)	<0.01	4.53 (2.63,7.83)	<0.01	5.7 (2.9,11.19)	<0.01
Q3	12.41 (6.64,23.19)	<0.01	4.93 (2.54,9.55)	<0.01	4.66 (1.98,10.96)	<0.01

Crude model: adjusted for none. Model 1: adjusted for age and gender. Model 2: adjusted for age, gender, BMI, FBG, ALT, AST, GGT, LDL-C, TG, and Cr.

**Figure 4:**
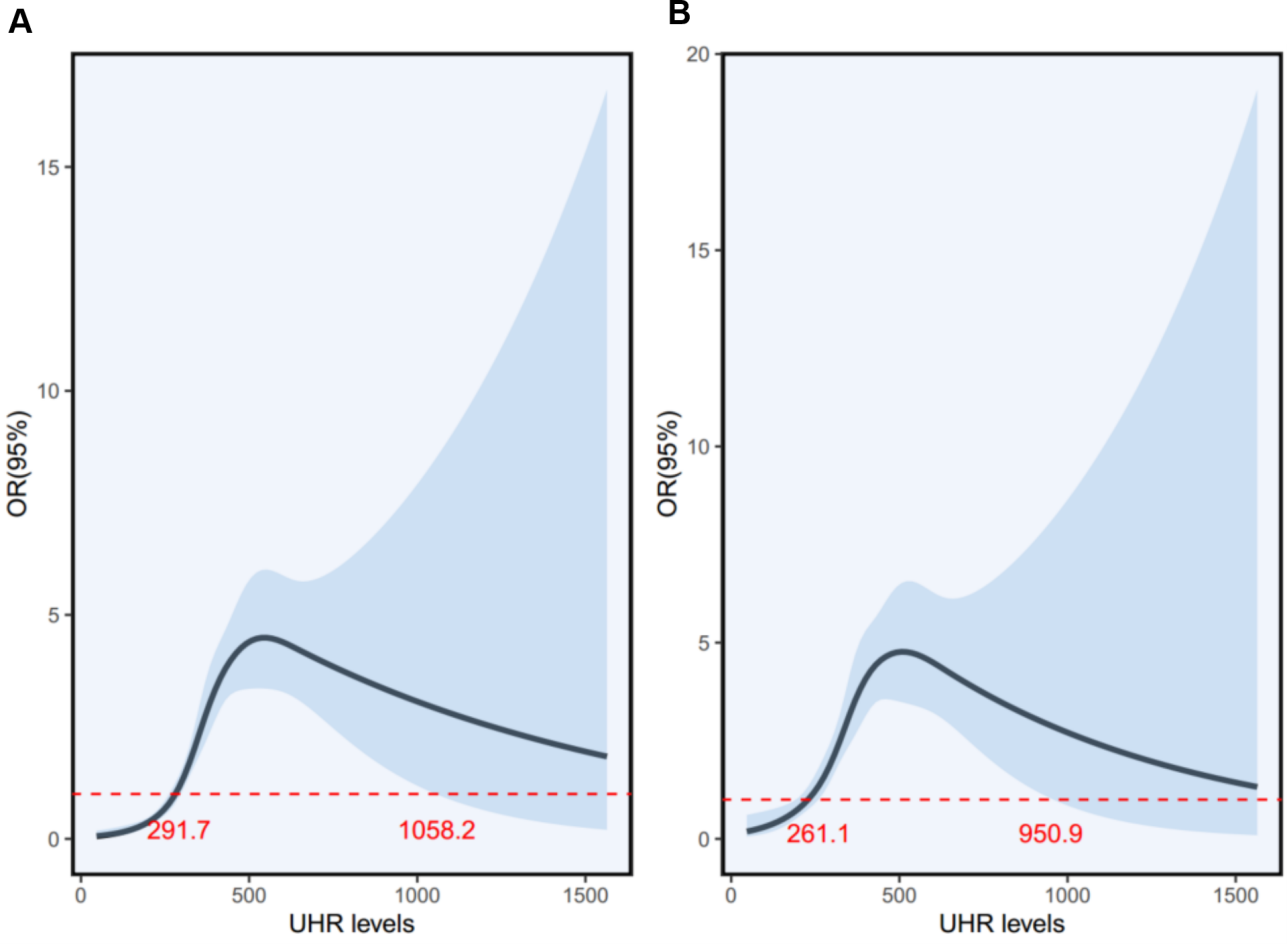
The unadjusted **(A)** and adjusted **(B)** ORs for MASLD by UHR in children with obesity. UHR, uric acid-to-high-density lipoprotein cholesterol ratio; MASLD, metabolic dysfunction-associated steatotic liver disease; ORs, odds ratios.

**Figure 4** presents the restricted cubic spline curves illustrating the association between baseline UHR levels and the risk of MASLD. The left panel shows the unadjusted odds ratio (OR) curve, revealing a non-linear relationship with significant risk increases between two critical points of UHR level, 291.7 and 1058.2, where the OR’s lower limit of the 95% confidence interval (CI) equals 1.0. The right panel shows the OR curve adjusted for age, gender, BMI, FBG, ALT, AST, GGT, LDL-C, TG, and Cr, demonstrating a similar non-linear relationship with critical points at 261.1 and 950.9. The area between the two critical points indicates a higher risk of MASLD as UHR levels increase. Notably, within the range of approximately 300 to 900, the OR values and their 95% CIs are consistently greater than 1, indicating an increased risk of MASLD.

### ROC analysis of the predictive value of UHR for MASLD

**Figure 5:**
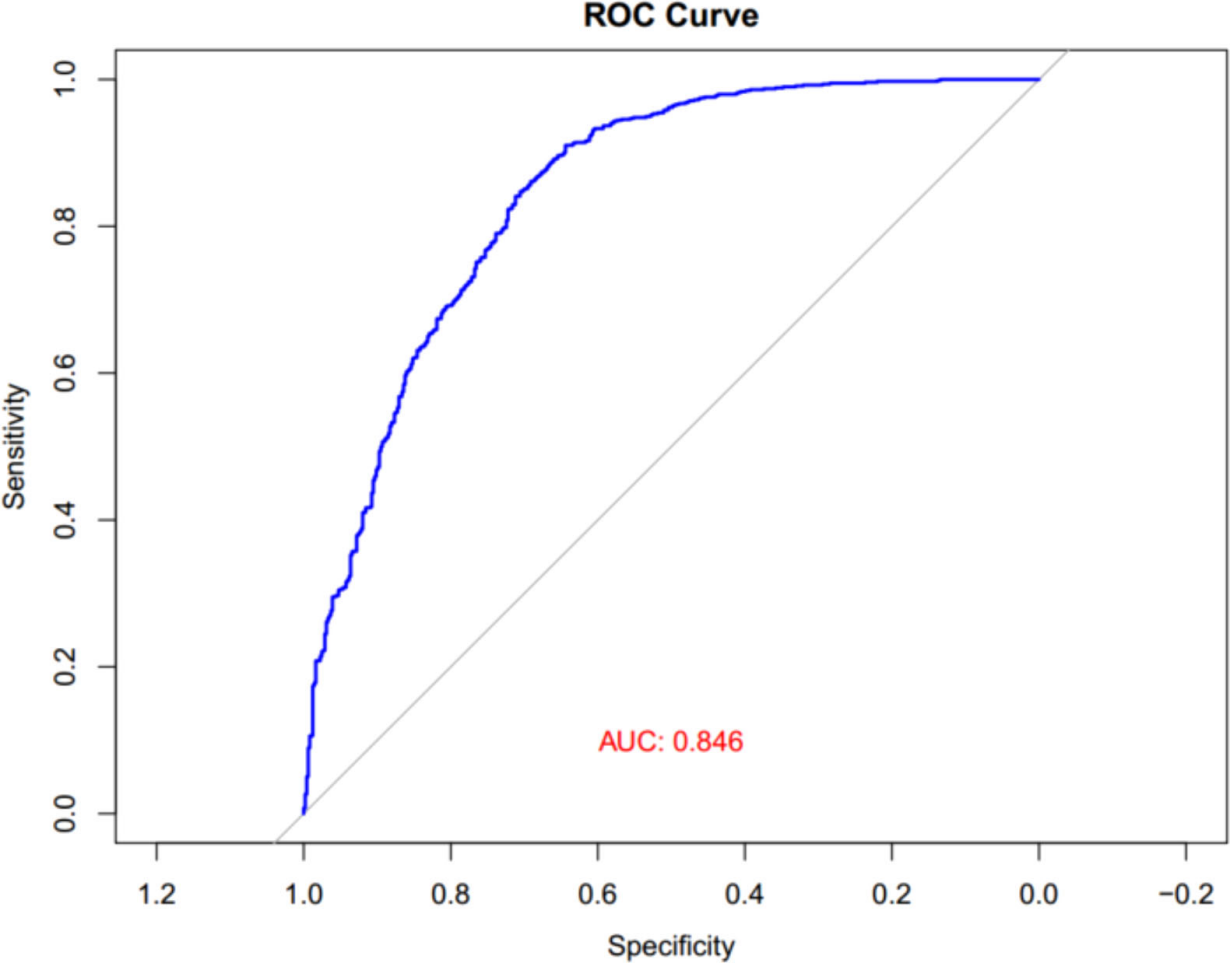
ROC curves of UHR for MASLD in children with obesity. UHR, uric acid-to-high-density lipoprotein cholesterol ratio; MASLD, metabolic dysfunction-associated steatotic liver disease; ROC, receiver operator characteristic.

Finally, ROC analysis was performed to assess the diagnostic significance of the UHR for MASLD in children with obesity. As illustrated in **Figure 5**, the area under the curve (AUC) for UHR in the ROC analysis was 0.846 (95% CI: 0.823–0.869).

## Discussion

The global burden of MASLD parallels the increase in obesity rates across the world. In recent years, the incidence of MASLD in obese children in China has risen sharply. In this study, the total prevalence of MASLD reached 61.76% in children with obesity in Beijing. A recent retrospective study in Hangzhou that enrolled 3216 children reported a similarly high prevalence of MASLD, with 1915 obese cases (59.55%) being MASLD. Another cross-sectional study that recruited 844 children in Changsha also reported a high prevalence rate of MASLD in overweight/obese children (38.2%). These studies indicate a severe situation of MASLD among obese children in China. However, the disease often has an insidious onset, the natural progression during childhood is unclear, and there is a lack of non-invasive and reliable diagnostic methods, posing significant challenges for pediatricians in clinical practice. Given the major significance of early identification of MASLD for prognosis and the disadvantages of commonly recommended examination methods, this study analyzed the correlation between UHR and MASLD among children with obesity.

Our research provided evidence for the close association between UHR and MASLD in children with obesity. Firstly, we observed significantly higher UHR levels in obese children with MASLD compared to those without MASLD. In addition, the prevalence of MASLD increased progressively from the lowest tertile to the highest tertile of UHR. Secondly, logistic regression analysis showed that children in the highest tertile of UHR had higher risks for MASLD compared with those in the lowest tertile, suggesting that obese children with increased UHR levels are more likely to have MASLD. Additionally, the association between UHR and MASLD risks was independent of multiple confounding factors including age, gender, BMI, FBG, ALT, AST, GGT, LDL-C, TG, and Cr. Finally, the ROC analysis demonstrated that UHR might serve as a specific and sensitive marker for MASLD in children with obesity. To the best of our knowledge, our study is the first to explore the association between UHR and the risk of MASLD in children with obesity.

UA and HDL-C are the two most crucial metabolic variables that are altered in a fatty liver. Uric acid is the end product of purine metabolism. Increased UA levels lead to endothelial dysfunction, inflammation, oxidative stress, and insulin resistance, which are key factors in the development of MASLD (29). Mosca et al. reported that serum UA concentrations were independently associated with non-alcoholic steatohepatitis in children and adolescents with proven MASLD (30). HDL-C, known for its reversal cholesterol transportation function, exhibits excellent anti-inflammatory, anti-oxidative, anti-atherogenic, and insulin-sensitizing properties (31, 32). Previous evidence has confirmed that HDL-C is closely associated with MASLD (33, 34). UHR, which is calculated by dividing serum UA by HDL-C levels, has recently attracted increasing attention regarding its role in metabolic-related diseases. Zhou et al. conducted a cross-sectional study including 8817 American participants demonstrating the significant correlation between UHR and insulin resistance (17). They further conducted a study on 2545 patients with type 2 diabetes mellitus, which also showed a significant association between UHR and insulin resistance (18). Han and Ahari et al. demonstrated that UHR was a potential clinical marker of hypertension, especially in women (35, 36). Some studies have found that UHR was significantly correlated with the components of metabolic syndrome components and might serve as a novel predictor of metabolic syndrome (19, 37, 38). Our present research found a close association between UHR and MASLD. Our findings were in agreement with several previous studies. Zhang et al. performed a cross-sectional study among 6285 lean Chinese adults suggesting that UHR was significantly associated with MASLD and might serve as a reliable marker for MASLD in lean adults (39). Xie et al. revealed that UHR was independently related to an increased MASLD risk and the severity of liver steatosis in American individuals (26). The positive association between UHR and MASLD was also observed in type 2 diabetic adults without overweight or obesity (23). Our research presented for the first time that, in children with obesity, higher UHR was strongly and independently associated with an increased risk of MASLD.

In addition, the non-linear relationship analysis demonstrated a saturation effect on the relationship between UHR and MASLD risk. This phenomenon suggests that there is a threshold beyond which increases in UHR do not correspond to a proportional increase in MASLD risk. It may be attributed to the metabolic mechanisms involved in uric acid and HDL-C homeostasis. When UHR levels exceed a certain threshold, it is plausible that the body’s compensatory mechanisms reach a plateau, thereby attenuating the further increase in MASLD risk. High levels of uric acid are known to promote oxidative stress, insulin resistance, and hepatic lipid accumulation, while low HDL-C levels impair lipid clearance and increase hepatic fat storage. Once these processes reach a maximum response threshold, additional increases in UHR may not further amplify MASLD risk, leading to the observed saturation effect. In a population-based analysis conducted in 3766 American adults, the non-linear relationship analysis also demonstrated a saturation effect on the relationship between UHR and MASLD risk (23). These results highlight the need for further research to elucidate the underlying mechanisms and to refine risk assessment strategies.

There are some limitations in this study. Firstly, owing to the cross-sectional design of the study, the causality of UHR and MASLD could not be established. Secondly, as a single-center study, the conclusions cannot be generalized to the entire Chinese population. Thirdly, the study population consisted only of obese children, with no normal-weight controls. Fourthly, given that our study design was retrospective and aimed primarily at identifying associations between UHR and MASLD presence, we did not incorporate a continuous variable, such as controlled attenuation parameter (CAP) or magnetic resonance imaging-derived proton density fat fraction (MRI-PDFF) to quantify the severity of MASLD. Therefore, prospective, large-sample studies are needed in the future to address these limitations.

In conclusion, our study shows that in children with obesity, UHR is independently and positively correlated with MASLD. This study underscores the potential of UHR as a non-invasive biomarker for MASLD diagnosis in children with obesity, highlighting the need for further prospective studies to confirm these findings and establish clinical guidelines for using UHR in pediatric populations.

## Data Availability

The raw data supporting the conclusions of this article will be made available by the authors, without undue reservation.
